# Accuracy of optical biometry combined with Placido disc corneal topography for intraocular lens power calculation

**DOI:** 10.1371/journal.pone.0172634

**Published:** 2017-02-23

**Authors:** Giacomo Savini, Kenneth J. Hoffer, Piero Barboni, Nicole Balducci, Domenico Schiano-Lomoriello, Pietro Ducoli

**Affiliations:** 1 Fondazione G.B. Bietti IRCCS, Rome, Italy; 2 Stein Eye Institute, University of California, Los Angeles, CA, United States of America; 3 St. Mary’s Eye Center, Santa Monica, CA, United States of America; 4 Studio Oculistico d’Azeglio, Bologna, Italy; 5 Scientific Institute San Raffaele, Milan, Italy; The Chinese University of Hong Kong, HONG KONG

## Abstract

**Purpose:**

To investigate the accuracy of a new optical biometer for intraocular lens (IOL) power calculation in eyes undergoing cataract surgery.

**Methods:**

Consecutive eyes of patients undergoing cataract surgery with the same IOL model were enrolled in a prospective cohort study.

Axial length (AL) and corneal power were measured with an optical biometer based on optical low-coherence interferometry and Placido-disc corneal topography. IOL power was calculated with the Hoffer Q, Holladay 1 and SRK/T formulas. For each formula the lens constant was optimized in retrospect in order to achieve a mean prediction error (PE) of zero (difference between the predicted and the postoperative refraction). Median absolute error (MedAE) and percentage of eyes with PE ±0.50 D were calculated.

**Results:**

Seventy-four eyes of 74 cataract patients were enrolled. The MedAE was 0.25 D with all formulas. A PE within ±0.50 D was obtained in 89.04% of cases with the Hoffer Q and SRK/T formulas, and in 87.67% of cases with the Holladay 1 formula.

**Conclusions:**

The optical biometer investigated in the present study provides accurate measurements for IOL power calculation.

## Introduction

Intraocular lens (IOL) power calculation has gained great interest in the era of refractive cataract surgery. In the last few years, a growing number of manufacturers have developed instruments able to measure the clinical parameters needed to calculate the IOL power. Since the IOLMaster (Carl Zeiss Meditec, Jena, Germany) was first introduced in 1999, the technology has undergone continuous evolution and a number of other instruments have been released, including the Lenstar LS900 (Haag-Streit, Köniz, Switzerland), the Aladdin (Topcon Europe, Visia Imaging, San Giovanni Valdarno, Arezzo, Italy), the AL-Scan (Nidek Co. Ltd., Gamagori, Japan), the Galilei G6 (Ziemer, Port, Switzerland), the OA-2000 (Tomey, Nagoya, Japan), the Argos (Movu, Santa Clara, CA), and the Pentacam AXL (Oculus, Wetzlar, Germany). These instruments use different technologies to measure axial length (AL), keratometry (K), anterior chamber depth (ACD) (corneal epithelium to lens,) and other values including lens thickness. However, little is known about the accuracy of the devices for IOL power calculation, with the exception of the IOLMaster [1–8], which was the first to be marketed, and the Lenstar LS900 [2, 5, 8–10]. Most papers, in fact, deal with agreement between instruments for certain measurements or their repeatability and reproducibility, but surprisingly the main purpose for which they were developed, i.e., IOL power calculation, has received little attention [11].

This study was designed to assess the accuracy of the Aladdin for IOL power calculation, with special attention to the final outcome, i.e. the postoperative refraction.

## Methods

This was a prospective cohort study. Before being included in the study, all patients were informed of its purpose and gave their written consent. The study methods adhered to the tenets of the Declaration of Helsinki for the use of human participants in biomedical research. The research protocol was reviewed and approved by the ethics committee of GB Bietti Foundation, Rome, Italy.

### Patients and surgery

All consecutive patients having cataract surgery in a single practice between January 2014 and April 2016 were prospectively enrolled. Phacoemulsification was performed by two surgeons (G.S. and P.B.) using the same technique through a temporal near-clear 2.2 mm incision under topical anesthesia. Since different IOL models (including toric and multifocal IOLs) were used and constant optimization should be carried out separately for each IOL model [12], we decided to investigate only the patients who received the most commonly implanted IOL model (i.e., the Acrysof SN60WF IOL, Alcon Laboratories Inc., Fort Worth, TX).

Exclusion criteria were: prior corneal or intraocular surgery, keratoconus and any other corneal disease, contact lens usage during the last month, and postoperative best corrected visual acuity lower than 0.8 (20/25) for any reason.

### Instrumentation

The Aladdin combines an optical biometer and a Placido-ring topographer. It can measure AL, K, anterior chamber depth (ACD), corneal diameter and pupil diameter. Its repeatability and reproducibility have already been assessed [13, 14]. Optical biometry relies on optical low-coherence interferometry (OLCI), based on an 830 nm super-luminescent diode that is used to measure the AL of the eye. Corneal topography is based on the reflection of 24 Placido disk rings with a diameter of 8 mm. However, K values are derived not from simulated K (SimK), but rather from automated keratometry, in which the values are generated from the reflection of 4 dedicated Placido rings (for a total of 1024 points) with a diameter ranging between 2.4 mm and 3.4 mm. Corneal curvature data are converted to corneal power by means of the standard keratometric index (1.3375). In this study, the average of the flattest and steepest K was recorded and subsequently used for IOL power calculation. Each patient underwent three acquisitions performed with the optical biometer combined with Placido disc topography, for a total of 18 AL measurements and 3 K measurements. The data provided in the final printout represented the average of the measurements of each parameter.

For comparative purposes, K was also measured with a validated Placido disk–based corneal topographer, the Keratron (Optikon 2000 Spa) [15, 16], which projects 26 rings onto the cornea to calculate the SimK (at the diameter of 3.0 mm), and AL was also measured by using Ocuscan ultrasound (US) immersion biometry (Alcon Laboratories, Inc. Ft. Worth, TX). While the Aladdin is a large-cone Placido topographer, the Keratron is a small-cone system. The combination of SimK by Keratron and AL measurement by US immersion biometry was chosen as it had provided very good results in previous studies [17–19].

### IOL power calculation and constant optimization

Preoperatively, IOL power was calculated using the Hoffer Q, Holladay 1 and SRK/T formulas [20–22]. A final evaluation was performed by assessing the subjective spherical equivalent refractive outcomes 1 month postoperatively, which is when refractive stability can be expected with small-incision clear cornea surgery and this type of IOL [23–25]. The distance from the patient’s eye to the acuity chart was 6 m. To calculate the prediction error in refraction, the refraction was subtracted from the predicted refraction (based on the IOL actually implanted) according to each formula. The mean prediction error (ME), the median absolute error (MedAE) and the mean absolute error (MAE) were calculated, as well as the percentage of eyes with a prediction error (PE) within ±0.50 D [12].

Predictions made using the Hoffer Q, Holladay 1, and SRK/T formulas were optimized in retrospect by adjusting the lens constants to give a PE of zero for the average case, according to the method described by Hoffer [20, 26] and Olsen [4]. As a result, it was possible to evaluate the statistical error as representing the optimum PE, rather than offset errors related to incorrect lens constants or systematic errors in the measuring environment. Optimization was performed using Hoffer Programs software (version 2.5).

### Statistical analysis

A paired t-test (for parametric data) and Wilcoxon matched pairs test (for non-parametric data) were performed to compare the mean values of corneal power, AL, and the absolute value of the PE. The chi-square test was used to compare the percentage of eyes with a PE within ±0.50 D. The variance ratio test (F-test) was used to compare the variances of the PE. A p value less than 0.05 was considered statistically significant. All statistical tests were performed using Graphpad Instat for Windows (version 3.10, Graphpad Software) and MedCalc (version 12.3.0.0, MedCalc Software). For patients who had bilateral surgery, only the first eye operated on was considered for statistical analysis.

Based on power and sample size calculations performed using the PS program (version 3.0.12), it was estimated that a sample size of 35 eyes would be necessary to detect a difference in MedAE of 0.05 D with a power of 95% at a significance level of 5%, given a within-subject standard deviation for simulated K equal to 0.10 D [14].

## Results

Out of 303 eyes operated on during the recruitment period, 74 eyes of 74 patients (mean age: 74.1 ±8.5 years; females: 42) were analyzed. In one case (1.4%) AL could not be measured by the optical biometer because of lens opacities. Therefore, only 73 eyes were investigated. Their clinical features are shown in Table 1.

**Table 1:** 

	Optical biometry + corneal topography	Ultrasound immersion biometry + corneal topography	Paired t-test
Corneal power	43.50 ±1.25 D (range: 40.00–46.07)	43.41 ±1.27 D (range: 39.80–46.37)	P = 0.0017
Axial length	24.04 ±1.29 mm (range: 21.09–30.17)	23.94 ±1.27 mm (range: 20.96–29.84)	P < 0.0001
Short eyes (axial length <22 mm)	2 (2.7%)	2 (2.7%)	N/A
Long eyes (axial length <26 mm)	5 (6.6%)	4 (5.4%)	N/A
Target refraction	-0.47 ±1.07 D (range: -3–0)		
Implanted IOL power	20.66 ±3.25 D (range: 10–35)		

Clinical features of the study eyes. D = diopters.

**Fig 1:**
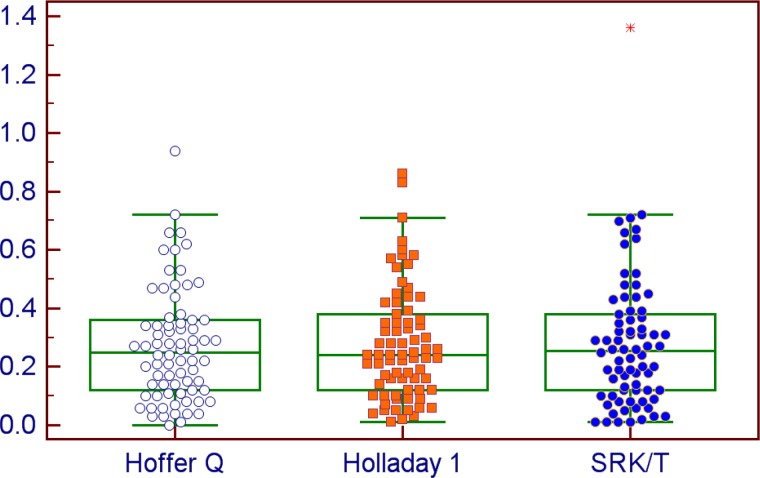
Mean absolute prediction error by the Hoffer Q, Holladay 1 and SRK/T formulas using the Aladdin measurements.

With the optical biometer, the ME was always ≤0.02 D as a result of constant optimization. The MedAE was 0.25 D for all formulas and the rate of eyes with a PE ≤ 0.50 D ranged between 87.67 and 89.04% (Table 2). With the Hoffer Q and Holladay 1 formulas, all eyes had a PE ≤ 1.00 D. Fig 1 shows the distribution of the absolute PE with the three formulas using the K and AL measurements by the optical biometer.

**Table 2:** 

	Hoffer Q (Aladdin)	Hoffer Q (SimK+US)	Holladay 1 (Aladdin)	Holladay 1 (SimK+US)	SRK/T (Aladdin)	SRK/T (SimK+US)
Mean optimized constant	5.69	5.45	1.91	1.68	119.11	118.77
Mean PE	0.02 ±0.33 D	0.01 ±0.37 D	0.02 ±0.34 D	0.01 ±0.36 D	0.00 ±0.36 D	-0.01 ±0.39 D
MedAE	0.25 D	0.16 D	0.25 D	0.19 D	0.25 D	0.19 D
MAE (±SD)	0.27 ±0.19 D	0.26 ±0.25 D	0.28 ±0.19 D	0.27 ±0.24 D	0.28 ±0.23 D	0.28 ±0.27 D
PE Variance	0.04 D	0.07 D	0.04 D	0.06 D	0.05 D	0.07 D
Eyes with PE within ±0.25 D	50.68%	71.23%	56.16%	65.75%	53.42%	61.64%
Eyes with PE within ±0.50 D	89.04%	86.30%	87.67%	87.67%	87.67%	86.30%
Eyes with PE within ±1.00 D	100.00%	97.26%	100.00%	98.63%	98.63%	95.89%

Refractive outcome predictability of IOL power calculation using the Aladdin optical biometer. D = diopters; MedAE = median absolute error; MAE = mean absolute error; SD = standard deviation; PE = prediction error.

IOL power calculation based on AL measured by immersion US biometry and K measured by Placido-disc corneal topography provided results that were close to those obtained with the Aladdin biometer (Table 2). Notwithstanding slightly different mean K and AL values, no statistically significant differences were detected between the two techniques when comparing the absolute error and the percentage of eyes with a PE within ±0.50D. However, with all formulas the Aladdin yielded a slightly lower PE variance; according to the F-test, such a difference was statistically significant for the Hoffer Q (p = 0.014) and Holladay 1 (p = 0.039) formulas.

## Discussion

Our data suggest that the AL and K measurements provided by the optical biometer combined with Placido corneal topography can lead to accurate IOL power calculation when entered into third-generation IOL power formulas. With all tested formulas we obtained a PE within ±0.50 D higher than 87%, which is well above the 55% value established as the benchmark standard by the National Health Service of the United Kingdom [27]. Our results seem slightly better than those previously reported by Kaya et al. using the same optical biometer, as they obtained 71% of eyes with a PE within ±0.50 D [28]. It is not clear if these authors optimized the constants in retrospect to get a mean zero PE, so any comparison should be done with caution.

When compared to other optical biometers, the instrument investigated in the present study showed similar or better outcomes (Table 3). More specifically, our results were better than those reported by different authors using the Lenstar and the same IOL model [2, 8, 9], and similar to those reported by Reitblat et al. using the Lenstar and IOLMaster and the multifocal version of the same IOL [5]. The refractive accuracy was better than that reported by a number of authors who used the IOLMaster [2, 6, 8, 9]. Differences with respect to the outcomes of these instruments may be related to many factors, including the different technology used to measure corneal curvature (Placido disk-based corneal topography with the Aladdin and automated keratometry for the IOLMaster and Lenstar), different surgical techniques by the authors of previous papers (e.g., different size in the diameter of the capsulorexhis), and different criteria of enrollment (in our study we included only eyes with VA ≥0.8, whereas in most papers VA ≥0.5 was sufficient). A comparison to the landmark paper by Aristodemou et al. [1], who performed biometry with the IOLMaster on more than 8,000 eyes, is difficult because these authors did not report the total results of their sample, but stratified them according to the AL. According to their data, in the subgroups with medium AL (between 22.00 and 26.49 mm) the rate of eyes with a PE within ±0.50 D was lower than 80% in almost all cases. Although it is not possible to make a direct comparison, since they used IOL models differing from our own, the results of our study appear to be more accurate.

**Table 3:** 

	Instrument	IOL model	Sample size	Formula	MedAE (D)	MAE (D)	PE ≤ 0.50 D
Present study	Aladdin	Acrysof SN60WF	73	Hoffer Q	0.25	0.27	89.04%
Cooke 2016	IOLMaster	Acrysof SN60WF	1079	Barrett	0.25	0.31	80.6%
Hoffer 2010	IOLMaster	Acrysof SN60WF	50	Haigis	N/A	0.46	56%
Olsen	IOLMaster	AcrySof MA60AC	461	Olsen	N/A	0.43	62.5%
Reitblat 2015	IOLMaster	Restor SN6AD1	73	SRK/T	N/A	0.24	91.78%
Srivannaboon 2013	IOLMaster	PY-60AD (Hoya)	163	Hoffer Q	N/A	0.32	68%**
Cooke 2016	Lenstar	Acrysof SN60WF	1079	Olsen	0.22	0.28	83.7%
Hoffer 2010	Lenstar	Acrysof SN60WF	50	Haigis	N/A	0.45	58%
Hoffmann 2013	Lenstar	3 IOL models*	308	Holladay 1	0.26	0.31	79.2%
Hoffmann 2013	Lenstar	Acrysof SN60WF	82	Holladay 1	0.25	0.31	N/A
Reitblat 2015	Lenstar	Restor SN6AD1	73	SRK/T	N/A	0.23	91.78%

Accuracy of biometric measurements for intraocular lens power calculation with different optical biometers and formulas (only the results of the best formula are shown).

* Acrysof SN60WF (Alcon), iMics1 (Bausch + Lomb), Tecnis (Abbott Medical Optics).

** the percentage refers only to eyes with an axial length between 22 and 24.5mm.

Several studies compared optical biometry and immersion US biometry. Most did not report any statistically significant difference between the two techniques [7, 29, 30]. Other authors found better results with optical biometry, but did not optimize constants for immersion US biometry, so that their results cannot be considered reliable [31, 32]. Our study supports the concept that optical biometry and US immersion biometry have similar accuracy. The slight differences of K and AL measurements could be balanced by appropriate constant optimization.

The optimized constants for the Aladdin in our sample (Hoffer Q = 5.69; Holladay 1 = 1.91; SRK/T = 119.11) are quite close to those reported by the ULIB website (http://ocusoft.de/ulib/c1.htm, accessed August 28, 2016) for the same IOL model and the IOLMaster (Hoffer Q = 5.64; Holladay 1 = 1.84; SRK/T = 119.00). The slight difference may be related to the fact that the optical biometer investigated in this study provides slightly different corneal powers than the IOLMaster according to the two studies that compared these biometers [33,34].

This study has some limitations. First, we did not use other optical biometers to calculate the IOL power in the same sample, so that any direct comparison is not possible. Second, the percentage of short and long eyes in our sample was relatively low (<10%): this may partially explain the excellent results obtained with the Aladdin, as IOL power calculation is known to be more accurate in medium eyes. Including a higher percentage of short and long eyes would likely result in less accurate refractive outcomes. Third, we did not investigate the results with other IOL designs, which may influence the refractive outcome [35]. Forth, we did not take advantage of the possibility offered by the Aladdin, i.e. the availability of a Placido disk-based corneal topography, which enables us to measure corneal asphericity and potentially exploit this feature when calculating IOL power [36]. Fifth, we did not correlate the PE and the axial length, but this would be the aim of a different kind of study, focused on the IOL power formulas rather than on the device measurements.

In conclusion, our data show that the Aladdin optical biometer is a reliable instrument for accurately calculating the IOL power in unoperated eyes undergoing cataract surgery.
