# Correction: Accuracy of optical biometry combined with Placido disc corneal topography for intraocular lens power calculation

**DOI:** 10.1371/journal.pone.0175145

**Published:** 2017-03-29

**Authors:** Giacomo Savini, Kenneth J. Hoffer, Piero Barboni, Nicole Balducci, Domenico Schiano-Lomoriello, Pietro Ducoli

There is an error in the caption for Fig 1. Please see the complete, correct Fig 1 caption here.

**Fig 1:**
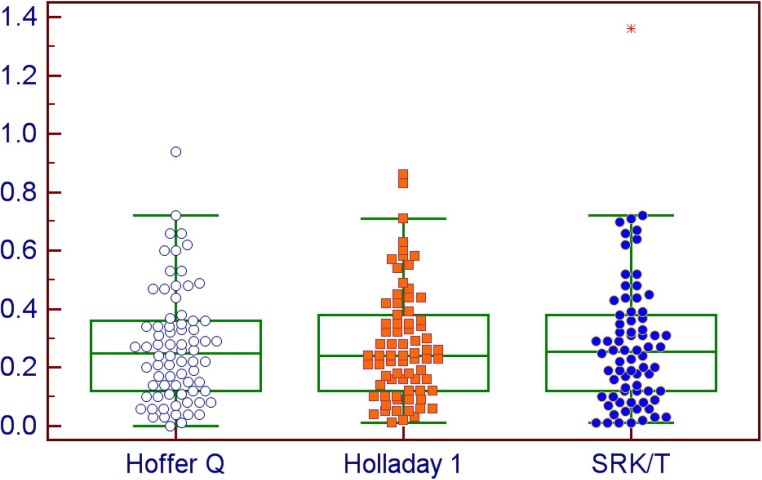
Median absolute prediction error by the Hoffer Q, Holladay 1 and SRK/T formulas using the Aladdin measurements.
